# Computer simulations of the mouse spermatogenic cycle

**DOI:** 10.1242/bio.20149068

**Published:** 2014-12-12

**Authors:** Debjit Ray, Philip B. Pitts, Cathryn A. Hogarth, Leanne S. Whitmore, Michael D. Griswold, Ping Ye

**Affiliations:** 1 School of Molecular Biosciences, Washington State University, PO Box 647520, Pullman, WA 99164, USA; 2 Biological Systems Engineering, Washington State University, Pullman, WA 99164, USA; 3 School of Electrical Engineering and Computer Science, Washington State University, Pullman, WA 99164, USA

**Keywords:** Spermatogenic cycle, Spermatogenesis, Germ cell, Mouse, Agent-based model, Simulation

## Abstract

The spermatogenic cycle describes the periodic development of germ cells in the testicular tissue. The temporal–spatial dynamics of the cycle highlight the unique, complex, and interdependent interaction between germ and somatic cells, and are the key to continual sperm production. Although understanding the spermatogenic cycle has important clinical relevance for male fertility and contraception, there are a number of experimental obstacles. For example, the lengthy process cannot be visualized through dynamic imaging, and the precise action of germ cells that leads to the emergence of testicular morphology remains uncharacterized. Here, we report an agent-based model that simulates the mouse spermatogenic cycle on a cross-section of the seminiferous tubule over a time scale of hours to years, while considering feedback regulation, mitotic and meiotic division, differentiation, apoptosis, and movement. The computer model is able to elaborate the germ cell dynamics in a time-lapse movie format, allowing us to trace individual cells as they change state and location. More importantly, the model provides mechanistic understanding of the fundamentals of male fertility, namely how testicular morphology and sperm production are achieved. By manipulating cellular behaviors either individually or collectively *in silico*, the model predicts causal events for the altered arrangement of germ cells upon genetic or environmental perturbations. This *in silico* platform can serve as an interactive tool to perform long-term simulation and to identify optimal approaches for infertility treatment and contraceptive development.

## INTRODUCTION

The spermatogenic cycle delineates the periodic development of male germ cells in a cross-section of the seminiferous tubule. The cycle has a fixed schedule that is specific to each species (França et al., 1998). In the mouse, one cycle lasts for 8.6 days and is divided into 12 stages based on the distinctive associations of germ cells in a cross-section (Ahmed and de Rooij, 2009; Oakberg, 1956b; Oakberg, 1957). It takes four cycles, equivalent to 35 days, for spermatogonial stem cells to undergo many differentiation steps needed to produce spermatozoa. During the process, germ cells gradually migrate from the tubule basement membrane toward the lumen before being released into the lumen (Oakberg, 1956a). Understanding the temporal–spatial dynamics of the spermatogenic cycle has important clinical implications because its disruption by genetic or environmental perturbations frequently leads to infertility or sub-fertility, and being able to regulate the process can provide new avenues to male contraceptives.

Traditionally, the spermatogenic cycle is examined by static imaging of seminiferous tubule cross-sections. Recent developments in dynamic imaging have enabled the visualization of live spermatogonial behaviors in the mouse (Nakagawa et al., 2010; Nel-Themaat et al., 2011; Stewart et al., 2009; Yoshida et al., 2007). However, prolonged imaging of the entire cycle remains impractical. More important, the molecular and cellular mechanisms underlying the complex dynamics of germ cells are poorly understood. In particular, many fundamental questions center on the periodic patterning of germ cells. This patterning results from multiple cellular behaviors including feedback regulation, mitotic and meiotic division, differentiation, apoptosis, and movement. Disruption in these cellular behaviors produces abnormal testicular morphology. In the absence of experimental techniques that enable individual cells to be followed throughout the spermatogenic cycle, a more profound understanding requires the assimilation of individual cellular behaviors into computational models that are quantitative and predictive.

A tractable *in silico* model will provide a unique approach for understanding and interpreting the arrangement of germ cells in the seminiferous epithelium and the continual production of spermatozoa. *In silico* models can be used to overcome the time-scale and spatial-scale limitations of live tissue imaging (Nakagawa et al., 2010; Nel-Themaat et al., 2011; Stewart et al., 2009; Yoshida et al., 2007). One such model is an agent-based model (ABM), a stochastic approach for studying the temporal–spatial dynamics of individual agents (Fallahi-Sichani et al., 2012; Grimm et al., 2005; Plikus et al., 2011). ABM consists of 1) an environment, 2) individual agents residing in the environment, 3) rules governing the behavior of agents and their interactions with other agents and the environment, i.e., the mechanisms, and 4) time scales of these behaviors and interactions.

Here, we report the development of a two-dimensional (2D) ABM to simulate germ cell development in a tubule cross-section over a time scale of hours to years. In the model, individual germ cells are discrete agents that are autonomous in making decisions and can adapt their decisions to the rapidly changing tubule environment. The global, system-wide testicular tissue pattern emerges from the local, mostly stochastic, individual-level cellular behaviors as visualized from time-lapse movies. Using this *in silico* platform, we then manipulate cellular behaviors either individually or simultaneously to understand why spermatogenesis occurs in a cyclic manner as a fundamental basis for male fertility. Further, we simulate the dynamic process leading to abnormal testicular morphologies that alter male fertility and predict the causal events. The model serves as an interactive tool to investigate the mechanisms that regulate germ cell arrangement within the seminiferous epithelium and the timing of sperm release. This approach may lay the foundation for identifying successful strategies for male infertility treatment and contraceptive development.

## MATERIALS AND METHODS

### Agent-based model

**Fig. 1.**
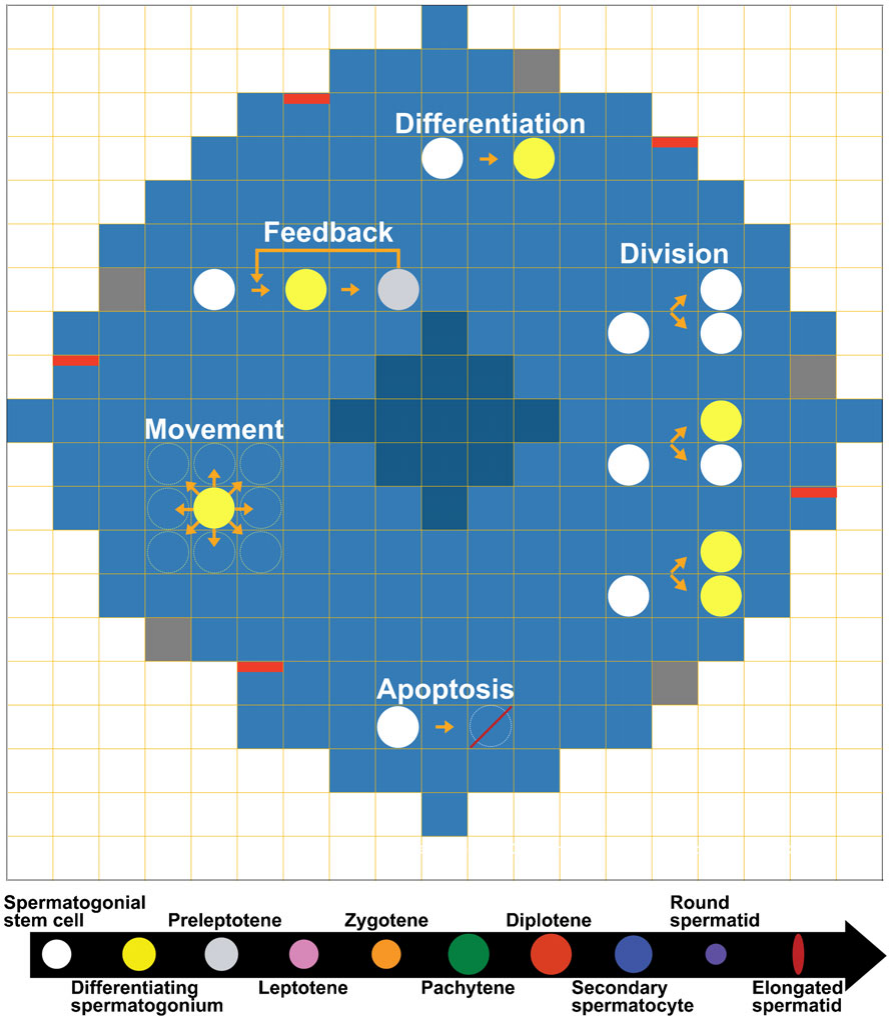
An ABM model to simulate the spermatogenic cycle of an adult mouse. Light-blue squares depict a cross-section of seminiferous tubule. Dark-blue squares represent the lumen of the tubule. Red bars indicate the areas close to the interstitium. Gray squares represent the space occupied by Sertoli cell nuclei. Germ cells of ten different types are color-coded. Rules including division, differentiation, apoptosis, movement, and feedback are based on known cellular behaviors.

We developed a 2D ABM to simulate germ cell dynamics on a cross-section of the seminiferous tubule in an adult mouse (Fig. 1). Testicular tissue patterns are achieved via a set of rules governing movement and interaction between cells and environment. The model has sufficient spatial and temporal resolution to allow observations at the level of individual cells.

#### Environment

A circular cross-section is captured in a regular grid. A circular lumen area is positioned in the middle of the cross-section, which is devoid of any cell types. Each micro-compartment is 13 µm by 13 µm, determined by the size of the largest germ cell type, pachytene. The radius of the cross-section is 14 micro-compartments, i.e., 180 µm; the radius of the lumen is 4 micro-compartments, i.e., 50 µm (Hogarth and Griswold, 2010; Nel-Themaat et al., 2011; Ray et al., 2012) (newly generated, see the Experiments subsection).

Sertoli cells are a type of somatic cells that provide structural and nutritional support for developing germ cells. The nucleus of a Sertoli cell is located at the basement membrane and the cytoplasm spans throughout the epithelium (Hogarth and Griswold, 2010). Although Sertoli cells are not treated as agents in our model, their nuclei occupy micro-compartments close to the basement membrane at equal angular locations, which are made inaccessible to germ cells. We consider ten Sertoli cells in one cross-section (Hogarth and Griswold, 2010; Nel-Themaat et al., 2011; Ray et al., 2012) (newly generated, see the Experiments subsection). Interstitial areas are spaces between tubules that house blood vessels. Five micro-compartments that are evenly spaced along the basement membrane are used to denote their proximity to the interstitium (Hogarth and Griswold, 2010; Nel-Themaat et al., 2011; Ray et al., 2012) (newly generated, see the Experiments subsection). These micro-compartments are accessible to germ cells.

#### Agents

Each cell is captured as a discrete agent. For simplicity, all cells are round in shape except for elongated spermatids, which are of oval shape. Diameters of ten germ cell types are estimated from fixed and live tissues and are given below (Ahmed and de Rooij, 2009; Bellvé et al., 1977a; Drumond et al., 2011; Hogarth and Griswold, 2010; Nel-Themaat et al., 2011; Ray et al., 2012) (newly generated, see the Experiments subsection): spermatogonial stem cell (9 µm), differentiating spermatogonium (10 µm), preleptotene (10 µm), leptotene (9 µm), zygotene (9 µm), pachytene (13 µm), diplotene (13 µm), secondary spermatocyte (11 µm), round spermatid (6 µm), and elongated spermatid (width  =  2 µm). Two round/elongated spermatids may co-occupy one micro-compartment, while a single micro-compartment can accommodate only one cell of any other types. Differentiating spermatogonia collectively represent A_1_, A_2_, A_3_, A_4_, In_m_, and B spermatogonia.

#### Rules

Each type of agent has unique behaviors, defined by four sets of rules. If simultaneous events occur for a cell, the simulation executes the events in the order of division, death, movement, and differentiation.

1. Division. Division generates an additional cell that occupies one of the eight neighboring micro-compartments around the parent cell. Division does not occur if space is unavailable. To represent a balanced self-renewal and differentiation at the population level, we implement asymmetric division for spermatogonial stem cells in the model, i.e., one cell divides to give rise to one stem cell (self-renewal) and one differentiating spermatogonium (differentiation). We further assume the asymmetric division depends on the appearance of preleptotenes. Experimental evidence shows that by 8 days postpartum (dpp) the endogenous retinoic acid (RA) signals cannot be overridden by exogenous RA (Raverdeau et al., 2012; Snyder et al., 2011; Sugimoto et al., 2012). Because 8 dpp correlates roughly to the first appearance of preleptotenes and RA is required for A to A_1_ spermatogonial transition (Morales and Griswold, 1987; Oakberg, 1956b; van Pelt and de Rooij, 1990), this observation suggests that spermatogonial differentiation may be triggered by RA from preleptotenes. In the model, additional three cell types undergo division. One differentiating spermatogonium divides to generate two daughter cells of the same type; the daughter cells inherit all their parent's properties to differentiate, divide, die, or move. One diplotene divides into two secondary spermatocytes, and one secondary spermatocyte divides into two round spermatids.
2. Death. With the exception of the spermatogonial stem cell, every cell type has a lifespan given in a range. We assume that the lifespan of spermatogonial stem cell is longer than its division time, thus division controls its population size. Other types of cells are removed from the system after reaching their respective lifespans.
3. Movement. Each cell can move into one of eight adjacent micro-compartments if they are unoccupied. Spermatogonial stem cells are attracted to the closest interstitium (Yoshida et al., 2007). Differentiating spermatogonia mainly move horizontally along the basement membrane. Other cells all tend to move toward the lumen. Every cell type has a position domain (Ahmed and de Rooij, 2009; Drumond et al., 2011; Hogarth and Griswold, 2010; Nel-Themaat et al., 2011) (newly generated, see the Experiments subsection), within which movement is allowed (supplementary material Table S1). If a cell fails to move because micro-compartments or a position domain is unavailable, it will re-try at the next time step.
4. Differentiation. Except for the following four cell types, all other cell types transition into the next stage by differentiation. As discussed above, the development of spermatogonial stem cell, diplotene, and secondary spermatocyte is dictated by division rules. Elongated spermatid is the terminal cell stage in the model; it is considered to become sperm once released into the lumen.

### Parameter estimation and initial condition

Kinetic parameters represent timers for cellular behaviors (supplementary material Table S2). Each timer decreases by one after each hour of the simulation. A cellular behavior is triggered once the timer reaches zero. Parameter values are obtained from dynamic imaging, irradiation experiments, and cell kinetic studies (Bellvé et al., 1977b; Jeyaraj et al., 2003; Klein et al., 2010; Nakagawa et al., 2010; Oakberg, 1956a; Oakberg, 1956b; Oakberg, 1957; Snyder et al., 2011; Yoshida et al., 2007). When values are unavailable, they are manually explored over a wide range and estimated based on the ability of the model to produce normal spermatogenic cycles (i.e., calibration). To account for biological variability and uncertainty in value estimation, the lifespan of each cell type follows a uniform distribution within a range. The initial condition is Stage I of the cycle, consisting of spermatogonial stem cells, differentiating spermatogonia, pachytenes, round and elongated spermatids; cells are randomly distributed in their respective position domains.

### Output from model simulations

Each time-step corresponds to one hour of real time. At each time-step, the simulation rules are executed and the state and position of each cell are updated accordingly. Each cell is assigned a unique lineage ID based on its parent–daughter relationship. The lineage is traced through the entire developmental path. We track the properties of each cell, including location and timers for future actions. These individual cell data are used to compute the count and position of each cell type and sperm. To visualize the results, image files are generated every hour during the simulation. Image frames are then combined into movies using iMovie (Apple Computer, Cupertino, CA).

### Parameter sensitivity analysis

The sensitivity analysis measures the variation in model output (e.g., cell number) upon the variation in model input (e.g., differentiation time). Latin hypercube sampling is used to sample multiple parameters simultaneously in a computationally efficient manner (Marino et al., 2008). Parameter values vary in a range of 0.5–1.5 fold of the baseline except for lifespan, which is varied between the maximum and 0.5 fold of the minimum. Parameters are uniformly sampled within these ranges for 300 times. Each sampled parameter set is run three times, and the average of the output is used to calculate partial rank correlation coefficient (PRCC) for parameter sensitivity. PRCC measures the nonlinear but monotonic relationship between one input parameter and one output variable after the removal of the linear effects of the remaining parameters. PRCC is in the range of 1 and −1 and associated with a p-value derived from Student's t-test.

### Computer simulations and visualization

The ABM model is implemented in C++, an object-orientated programming language. The agents (cells) are stored via a multi-map data structure, which is indexed by location and stores pointers to agent properties. Rules governing cellular behaviors are handled through a family of C++ classes. The code is developed on a Mac OS X 10.6.8 operating system and compiled using a GCC compiler. We use Qt 4.7.4 to generate graphical output and a user interface. Qt is a C++ framework for developing cross-platform applications with a graphical user interface.

### Experiments

All animal experiments were approved by Washington State University Animal Care and Use Committee and conducted in accordance with the guiding principles for the care and use of research animals of the National Institutes of Health. C57BL/6-129 mouse colonies were maintained in a temperature- and humidity-controlled environment with food and water provided *ad libitum*. Mice aged 60 dpp were euthanized by asphyxiation followed by cervical dissociation. Testes were collected and immediately placed in Bouin's fixative for five hours. Tissues were then dehydrated through a graded ethanol series and embedded in paraffin. Tissue sections of 3–5 µm were counterstained with hematoxylin for histological evaluation. Cross-sections of the seminiferous tubule were first staged based on the arrangement of cell types (Ahmed and de Rooij, 2009). For each of the 12 stages, 10 cross-sections were selected to count cell numbers of different types and to measure the position domain of each cell type using ImageJ (Schneider et al., 2012). The sizes of cross-section, lumen, and cells were also estimated from these images. The numbers of Sertoli cells and interstitial areas in a cross-section were obtained from these images as well.

## RESULTS

### A computer model to simulate the mouse spermatogenic cycle

To understand the temporal–spatial dynamics of germ cell development, we built an agent-based model to simulate the spermatogenic cycle of an adult mouse. The model depicts a circular cross-section of the seminiferous tubule in a regular grid. A circular lumen area is located in the middle of the tubule. Ten types of germ cells are depicted as individual agents: spermatogonial stem cell, differentiating spermatogonium, preleptotene, leptotene, zygotene, pachytene, diplotene, secondary spermatocyte, round spermatid, and elongated spermatid. Cellular behaviors of mitotic division, meiotic division, differentiation, apoptosis, movement, and feedback regulation are captured as rules in the model (Fig. 1). The rules are executed every one-hour of real time, and the state and position of germ cells are updated according to the rules. The execution of cellular behaviors depends on both kinetic rates and space constraints.

**Fig. 2.**
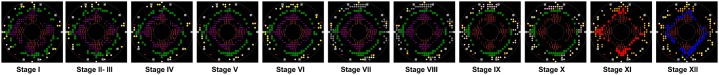
Snapshots of 12 stages from a simulated spermatogenic cycle. Germ cells of ten types are color-coded as defined in Fig. 1.

Using Stage I as the initial condition and baseline parameter values (supplementary material Table S2), the model readily reproduces 12 stages of the spermatogenic cycle (Fig. 2). Existing cell types from Stage I to VI are the same: spermatogonia, pachytenes, round and elongated spermatids. At Stage VII and VIII, preleptotenes are present, and new differentiating spermatogonia emerge from the next round of differentiation. At Stage IX, all round spermatids become elongated spermatids. Stage IX and X are characterized by the presence of spermatogonia, leptotenes, pachytenes, and elongated spermatids. At stage XI, zygotenes and diplotenes appear. At stage XII, diplotenes differentiate into secondary spermatocytes. The model is capable of simulating spermatogenic cycles for an infinite amount of time. Time-lapse simulation of four cycles is shown in supplementary material Movie 1, corresponding to 35 days in real time. The movie captures the developmental path of individual cells: how they differentiate, move, and interact with other cells. The cellular associations in the cross-section occur in a cyclic manner.

**Fig. 3.**
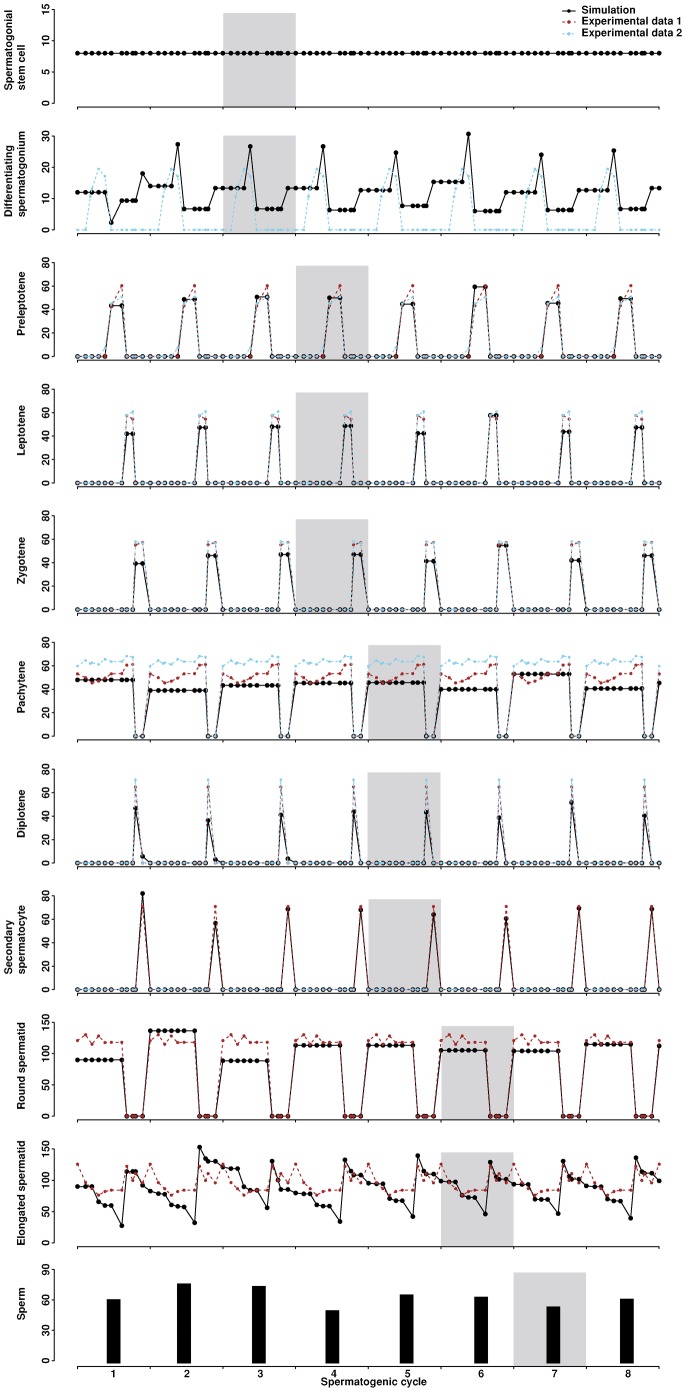
Numbers of germ cells and sperm over eight spermatogenic cycles. Stage I is the initial condition of the simulation. The average from three simulation runs is presented. Germ cells and sperm that originate from spermatogonia in the third cycle are shaded as an example to illustrate the developmental path. Experimental data for one spermatogenic cycle are replicated across eight cycles. Experimental data 1 are newly generated here, and experimental data 2 are from a previous report (Oakberg, 1956a).

The model quantifies germ cells of different stages throughout spermatogenic cycles (Fig. 3). The number of spermatogonial stem cells does not change over time, providing a constant source for differentiating germ cells. Downstream cells in our model, from differentiating spermatogonia to elongated spermatids, exhibit oscillations across cycles. Each cycle profile is unique and independent from the others, though the overall pattern is conserved across cycles. Simulation results for germ cells are comparable with two sets of experimental data: one set is our own data from this study by counting cells of different types in testicular cross-sections (see the Materials and Methods section), the other is from a previous report (Oakberg, 1956a). Elongated spermatids are considered as sperm once they enter the lumen. The model traces the sperm release to evaluate how testicular morphology affects the level of sperm production. Similar to the counts of differentiating germ cells, the sperm count fluctuates across cycles. This fluctuation is consistent with the nature of spermatogenesis, which is captured by the ABM approach. Note that it takes four cycles for spermatogonial stem cells to develop into mature elongated spermatids. For example, the spermatogonia in the third cycle give rise to the elongated spermatids in the sixth cycle and the sperm released in the seventh cycle (Fig. 3). We calculated the success rate of sperm production, i.e., the fraction of sperm released into the lumen compared to all sperm that could be produced from spermatogonial stem cells. The rate across 20 cycles is in the range of 17–28% with an average of 23%, consistent with estimates from experimental data of 20–50% (de Rooij and Russell, 2000). Cell counts of different types, especially those in successive developmental stages, are highly correlated, ranging from 0.67 to 0.99 (supplementary material Fig. S1).

The position profile illustrates how cells are associated and distributed at different stages of the spermatogenic cycle. For each cell type, the distance to the tubule center oscillates, but the overall pattern is conserved across cycles (supplementary material Fig. S2). Spermatogonia and preleptotenes remain near the basement membrane. Leptotenes, zygotenes, and pachytenes gradually move toward the lumen. Closer yet are diplotenes and secondary spermatocytes, shown briefly in stages XI and XII, respectively. Round spermatids have a tendency to migrate toward the lumen. Elongated spermatids stay closest to the lumen. The current model does not explicitly capture the transient, reverse movement of elongated spermatids towards the basement membrane and then back toward the lumen during Stage III–VI (Ahmed and de Rooij, 2009), although the time lapsed during this reverse movement is considered in the model.

### Cell lineage tracing

The model traces individual cells as they change state and location. We captured the developmental path of one spermatogonial stem cell over four spermatogenic cycles to visualize how many and which progenies originate from one parent cell (supplementary material Movie 2). A spermatogonial stem cell first generates one differentiating spermatogonium and one stem cell. The differentiating spermatogonium divides twice to produce four cells, of which one dies and three divide again and differentiate into preleptotenes. Although differentiating spermatogonia divide six times to produce preleptotenes (Hilscher et al., 1969), not all divisions are captured by our 2D model, which describes events occurring on a single cross-section. Among six preleptotenes, five become leptotenes and one reaches its lifespan and dies. Leptotenes differentiate into zygotenes, pachytenes, and diplotenes. Among five diplotenes, four complete the first meiotic division to produce secondary spermatocytes, while one fails to divide and eventually dies as diplotene. The eight secondary spermatocytes undergo the second meiotic division, producing 16 round spermatids. Three round spermatids die and 13 further differentiate into elongated spermatids. Among them, 10 enter the lumen to become sperm and the remainders die. If all cells produced from one spermatogonial stem cell were to become sperm, there would have been 32. Thus, the success rate of sperm production is 31% (10/32) for this single stem cell, similar to the earlier estimation based on one cross-section.

### Cellular behaviors important for spermatogenesis

**Fig. 4.**
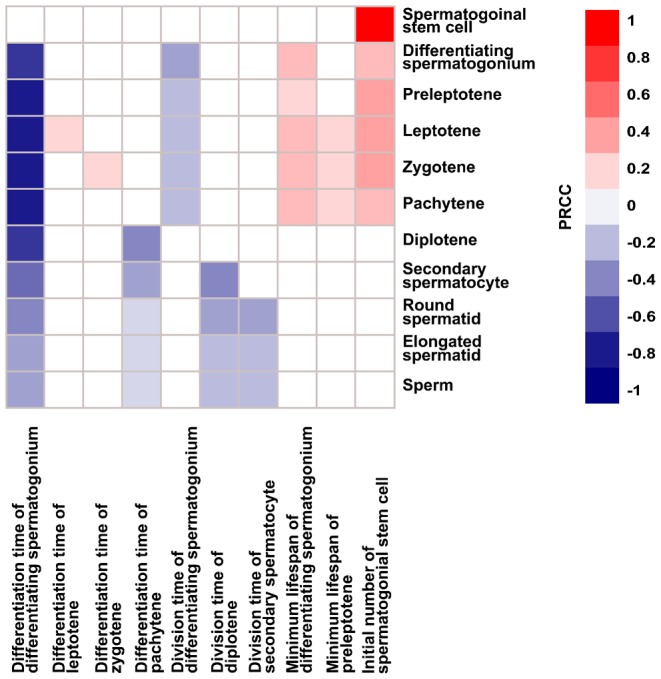
Parameter sensitivity analysis. Effects of parameters on germ cell numbers are investigated by simultaneously varying a total of 29 parameters. The range of variation is 0.5–1.5 fold of the baseline except for lifespan, which is varied between the maximum and 0.5 fold of the minimum. Random samples of each parameter are generated from the range with a uniform distribution. The sampling is performed 300 times to obtain 300 parameter sets. For each set, the average cell number is calculated across one spermatogenic cycle from three simulation runs. The correlation of model outputs (germ cell numbers) with each parameter is quantified by PRCC. A total of 10 parameters have significant PRCC values (p-value <0.005) with at least one germ cell type. Significant PRCC values are shown with red or blue colors. Insignificant PRCC values are not shown and marked with the white color.

When *in silico* models include parameters describing dynamic processes, it is critical to understand the role of each parameter in determining the output. Using the model, we can vary the type and timing of cellular behaviors in a variety of ways to explore system-wide outcomes on a scale that would be difficult or impossible by wet-lab approaches. We performed a parameter sensitivity analysis by simultaneously varying the 29 parameters controlling mitotic division, meiotic division, differentiation, apoptosis, movement, and the initial number of spermatogonial stem cells (supplementary material Table S2). The initial numbers of other cell types do not affect the system after the first four cycles. We identified 10 parameters that have significant influences on at least one cell type, i.e., with a significant PRCC value (Fig. 4; supplementary material Fig. S3).

The differentiation time negatively affects the population size of differentiating spermatogonia, but positively correlates with the population size of leptotenes and zygotenes, respectively. This difference reflects the quick entry to apoptosis when the differentiation of spermatogonia is delayed, while other cell types have a wider lifespan range resulting in their own accumulation. The differentiation time of either differentiating spermatogonia or pachytenes has negative effects on their respective downstream cells. Delayed differentiation causes the loss of spermatogonia and pachytenes in the model, thus fewer progress to the next stage. The division time of differentiating spermatogonia, diplotenes, or secondary spermatocytes negatively correlates with the number of their corresponding downstream cell types. The logic behind this phenomenon is similar to that for the differentiation time: cells postponing division undergo apoptosis instead. The minimum lifespans of differentiating spermatogonia and preleptotenes positively correlate with the population size of downstream cells, suggesting that longer lifespan ensures more progenies. The initial number of spermatogonial stem cells represents the size of the starting stem cell pool. This initial number has a perfect positive correlation with the population size of spermatogonial stem cells, and further influences the number of downstream cells.

Parameters important for sperm production are inferred from sensitivity analysis; they include the differentiation time of differentiating spermatogonia and pachytenes, and the division time of diplotenes and secondary spermatocytes. The carrying capacity of a cross-section, i.e., the maximum number of cells the section can hold, is fixed. Thus, cell division and movement are constrained by available space. Biologically, the space constraint corresponds to the total number of germ cells that a certain number of Sertoli cells can support. Therefore, sperm count does not keep increasing.

### Simulating spermatogenesis defects to understand underlying mechanisms

Both external and genetic perturbations can disrupt molecular pathways and alter germ cell associations. However, static images can only portray the outcome. Neither the dynamic process nor the precise mechanism leading to the outcome can be revealed. Using the *in silico* model, we can simulate the dynamics leading to abnormal testicular morphology and infer causal events by varying parameters. Case studies of vitamin A-deficient (VAD) mice and *Stra8*-deficient mice are provided below.

#### Case 1: VAD mice

Vitamin A deficiency blocks the transition from undifferentiated to differentiating spermatogonia, resulting in seminiferous tubules containing only spermatogonia and Sertoli cells. Administration of vitamin A removes the block and reinitiates spermatogenesis (Morales and Griswold, 1987; van Pelt and de Rooij, 1990). We tested all parameters and found that variations in each of six parameters – initial number and division of spermatogonial stem cell, differentiation time, division time, and lifespan of differentiating spermatogonium, and differentiation time of preleptotene – stop the transition to differentiating spermatogonia leading to the VAD phenotype (supplementary material Table S3). In our model, the asymmetric division of spermatogonial stem cells depends on the presence of preleptotenes at Stage VII and VIII. A variation in any of the six parameters causes either depletion or deficit of preleptotenes, thus disrupting the feedback regulation on spermatogonial differentiation from preleptotenes. Interestingly, four out of the six parameters are identified as key parameters from the PRCC analysis (Fig. 4), indicating that the feedback loop is key to maintaining the spermatogenic cycle. In reality, combinations of multiple parameter changes may be responsible for the VAD phenotype, which can be readily simulated using the model.

**Fig. 5.**
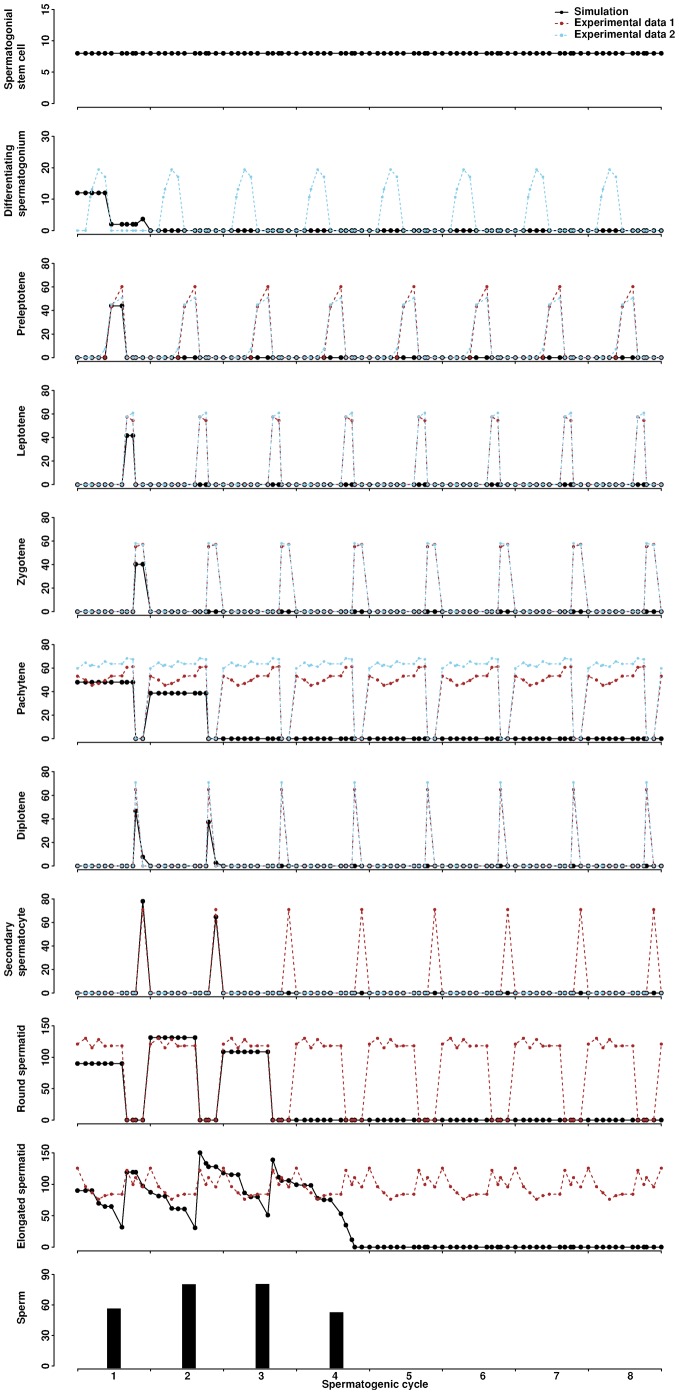
Simulation of the VAD mice. The preleptotene threshold required for asymmetric division of spermatogonial stem cells is changed from 5 (baseline value) to 50. A normal Stage I is the initial condition of the simulation. The average from three simulation runs is presented. Experimental data are the same as those in Fig. 3.

Starting from the normal Stage I of the cycle, we simulated the process of producing the VAD phenotype by raising the preleptotene threshold required for division of spermatogonial stem cells (Fig. 5; supplementary material Movie 3). Progressive germ cell depletion and cessation of spermatogenesis are observed. Spermatogonial stem cells are not affected. Differentiating spermatogonia, preleptotenes, leptotenes and zygotenes appear only during the first cycle. Pachytenes, diplotenes, and secondary spermatocytes appear for the first two cycles. Three peaks are observed for round and elongated spermatids and four peaks for sperm. Four spermatogenic cycles are required to eliminate all developing germ cells present at Stage I while spermatogonial stem cells remain in the tubule.

#### Case 2: *Stra8*-deficient mice

The *Stra8* gene encodes an RA-responsive protein. It is germ cell-specific and essential for meiotic entry (Anderson et al., 2008; Mark et al., 2008; Zhou et al., 2008). In the *Stra8*-deficient mouse, germ cell development is retarded at the preleptotene or leptotene stage; apoptotic cells are observed in tubules, which are rarely seen in wild-type testes (Anderson et al., 2008; Mark et al., 2008). To reproduce the *Stra8*-deficient phenotype, we evaluated all parameters and found that varying five parameters individually – differentiation time of preleptotene and leptotene, lifespans of preleptotene, leptotene, and zygotene – arrest spermatogenesis at either the preleptotene or leptotene stage (supplementary material Table S4). When differentiation is delayed or lifespan is shortened for preleptotenes, all preleptotene cells die before progressing to the next stage. Similar logic can explain stalled development at the leptotene stage. The immediate death of zygotenes also results in no cells beyond the leptotene stage.

**Fig. 6.**
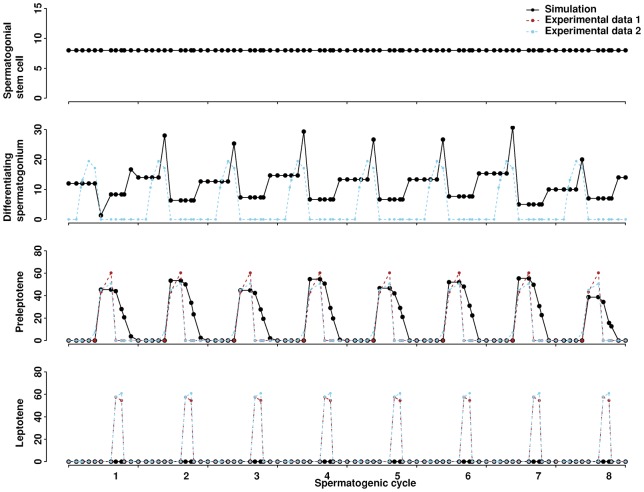
Simulation of the *Stra8*-deficient mice. Differentiation time of preleptotene is changed from 44 (baseline value) to 93 hours. The initial condition of the simulation is Stage I but with only spermatogonial stem cells and differentiating spermatogonia. The average from three simulation runs is presented. Experimental data are the same as those in Fig. 3.

We simulated spermatogenic cycles of the *Stra8*-deficient mouse by increasing the differentiation time of preleptotene (Fig. 6; supplementary material Movie 4). Spermatogenesis proceeds normally to preleptotene before arresting. The dynamics of spermatogonia are similar to the wild-type. Preleptotenes, however, exhibit slower decline at each cycle due to delayed differentiation. The number of preleptotenes is sufficient to trigger the asymmetric division of spermatogonial stem cells in the model, allowing the cycle to continue.

### Simulating contraceptive treatment to understand underlying mechanisms

The compound WIN 18,446 has been shown to completely and reversibly block spermatogenesis in men and function as a male contraceptive (Heller et al., 1961). Recent studies reveal that WIN 18,446-treated testes in mice and rabbits resemble those of the VAD phenotype, i.e., with seminiferous tubules containing only spermatogonia and Sertoli cells (Amory et al., 2011; Brooks and van der Horst, 2003). The likely cause is that WIN 18,446 inhibits conversion of retinal to RA, thereby blocking spermatogonial differentiation (Amory et al., 2011; Hogarth et al., 2011). Transient treatment of adult mice with this compound substantially affects the arrangement of germ cells within the seminiferous epithelium. For example, preleptotenes are absent one day after eight daily WIN 18,446 treatments; an injection of RA after the same treatment induces a thickened layer of round spermatids 26 days later (Hogarth et al., 2013). By manipulating cellular behaviors either individually or collectively *in silico*, the model can help to interpret and understand the altered arrangement of germ cells. Further, the model can simulate spermatogenic cycles upon or after treatment over a time scale of hours to years, providing a platform for evaluating the long-term effects of a reversible male contraceptive.

**Fig. 7.**
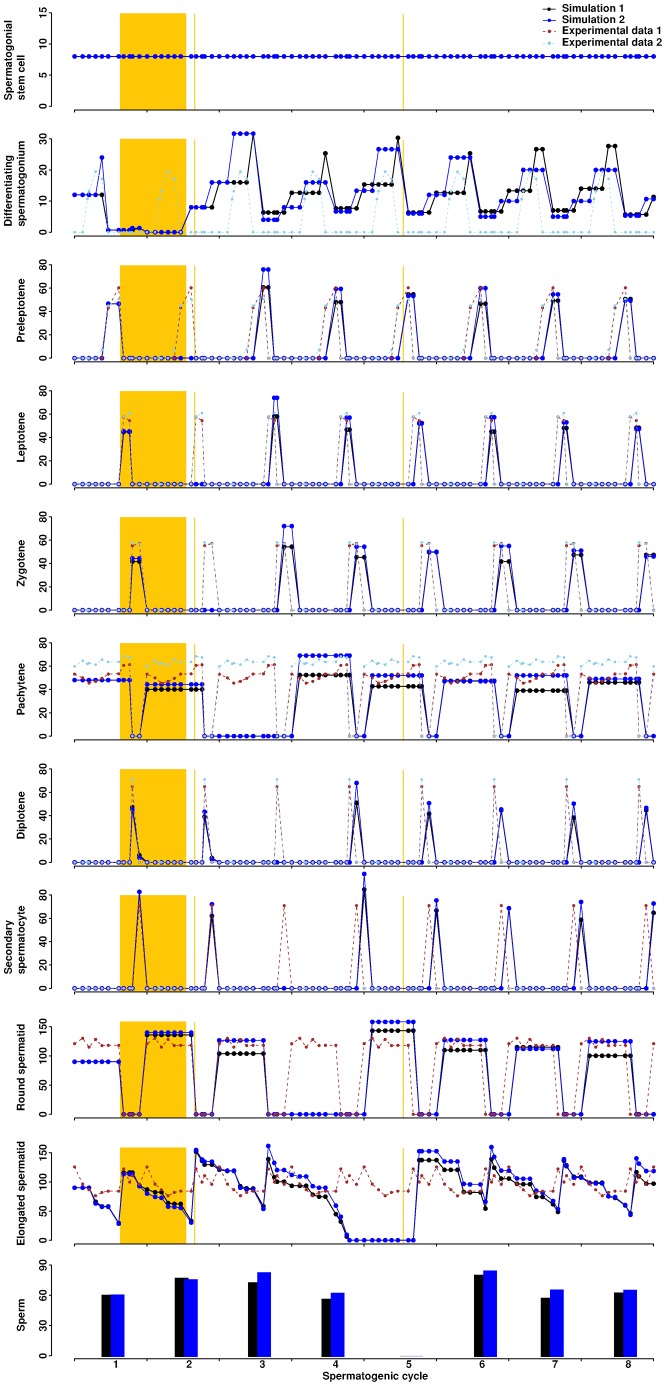
Simulation of an eight-day WIN 18,446 treatment in mice. Simulation 1 is generated by arresting the asymmetric division of spermatogonial stem cells for eight days starting from day five. Simulation 2 is generated by arresting the asymmetric division of spermatogonial stem cells for eight days starting from day five and accelerating the division time of differentiating spermatogonia from 88 (baseline value) to 68 hours. The orange rectangle marks the time window for arresting the asymmetric division. The first and second orange lines label 1 and 26 days post-treatment, respectively. A normal Stage I is the initial condition of the simulation. The average from three simulation runs is presented. Experimental data are the same as those in Fig. 3.

We simulated WIN 18,446 treatment by delaying the asymmetric division of spermatogonial stem cells in the model for eight consecutive days (Fig. 7; supplementary material Movie 5). The first characteristic change is that one cycle is missing upon treatment. This is a direct result of delaying the spermatogonial division for eight days. While the number of spermatogonial stem cells is unaffected, the development of all downstream cells is delayed by eight days. The second characteristic change is that the cycle immediately after the treatment becomes more efficient, resulting in a significantly larger number of progenies than in the wild-type simulation (p-value  =  0.02, one-tailed Welch two sample t-test for round spermatids at the 5^th^ cycle). Subsequent cycles, however, return to normal efficiency. The overall numbers of progenies are comparable between the treatment and the wild-type (p-value  =  0.45, one-tailed Welch two sample t-test for round spermatids over 20 cycles). These features may be attributed to the fact that more space becomes available in the epithelium after one absent cycle. Cellular behaviors such as division and movement have a higher probability to become successful, leading to a more potent cycle. Once the epithelium is repopulated with developing cells, cycle efficiency returns to normal. Therefore, the model predicts no long-term adverse effect of WIN 18,446 treatment. In addition, the model perfectly captures the testicular morphologies observed in treated mice (Hogarth et al., 2013). One day after the treatment, spermatogonia, pachytenes, and elongated spermatids are present in stages VIII–X. Preleptotenes or leptotenes, however, are missing. Twenty-six days (approximately three cycles) after the treatment, spermatogonia, preleptotenes, pachytenes, and a thickened layer of round spermatids are present in stage VII, but elongated spermatids are absent (Fig. 7).

We further explored whether changes in multiple parameters could result in similar morphologies. Because RA injection after WIN 18,446 treatment may increase spermatogonial proliferation, we simulated the spermatogenic cycles by arresting the asymmetric division of spermatogonia for eight days and accelerating the division of spermatogonia in the model (Fig. 7). Two characteristic changes are again observed: one cycle is missing upon treatment, and the cycle immediately following treatment becomes more efficient (p-value  =  0.01, one-tailed Welch two sample t-test for round spermatids at the 5^th^ cycle). Due to the increased proliferation of spermatogonia, more progenies are produced throughout cycles compared to those of the wild-type simulation (p-value  =  0.02, one-tailed Welch two sample t-test for round spermatids over 20 cycles). Theoretically, reducing death or increasing proliferation of any cell type would improve the efficiency of spermatogenesis.

## DISCUSSION

A fundamental feature of mammalian spermatogenesis is the continuous production of numerous spermatozoa throughout reproductive life. The spermatogenic cycle must be fine-tuned both temporally and spatially to ensure this high-level production. Although standard experimentation has revealed much about stages of spermatogenesis, prolonged imaging of live tissues remains impractical, precluding detailed analysis of the cycle dynamics. Computational models that collectively describe individual cellular behaviors through long-term simulations provide an essential complement to animal-based studies.

Computer programs have previously been developed to investigate spermatogenesis. One program called Stages tracks germ cell types and stages in the cycle of the seminiferous epithelium (Hess and Chen, 1992). The other program focuses on the behavior of spermatogonial stem cells in the niche, including division, differentiation, and movement (de Rooij and van Beek, 2013). Similar to our 2D computer model, both programs have the advantage of performing simulations over long periods of time as compared to experimental approaches. Our model, however, offers two major advantages as compared to the Stages program. First, our model is formulated based on mechanistic assumptions regarding germ cell behaviors and their interactions (i.e., feedback, division, differentiation, apoptosis, and movement); intrinsic behaviors of each cell are restricted by extrinsic space and neighboring cells, creating individual heterogeneity. The Stages program mainly depends upon the assumption of the cycle duration time and the frequency of each stage. Further, our model traces the state and position of individual cells, enabling the simulation of germ cell dynamics in a movie format. Sperm release is monitored to evaluate how testicular morphology affects the level of sperm production. The Stages program only reports the presence of germ cell types in a table format.

Notably, the main purpose of our model is not merely to achieve correlations with empirical data, but to understand the mechanisms that lead to normal and abnormal spermatogenic cycles. A computational model can reveal, in the same way as an animal model, key cellular behaviors by performing virtual perturbations and predicting system-wide outcomes. The advantage is that the *in silico* platform can be used to examine all possible mechanisms on a scale that would be difficult or impossible to achieve by experimental approaches. Our parameter sensitivity analysis identified that differentiation time and division time are the most important parameters influencing tubular morphology and sperm output. Indeed, the rigid timescale of differentiation and division is the key for generating staging patterns because a wide range of differentiation and division time creates asynchronous cohorts of germ cells and disrupts stages.

The model provides a platform for simulating dynamic processes and predicting cellular behaviors that lead to altered testicular morphologies upon genetic or external perturbations. The VAD study shows that removing the feedback loop from preleptotene to spermatogonial stem cells abolishes the cyclic pattern of spermatogenesis. This regulation can be considered as a combination of negative and positive feedback loops. Achieving preleptotene stage induces asymmetric division of spermatogonial stem cells in the model; differentiation reduces the number of spermatogonia (negative feedback) while self-renewal increases the number (positive feedback). Theoretical studies indicate that all biological oscillators are built around negative feedback loops; additional conditions are time delay (e.g., by positive feedback), sufficient nonlinearity, and comparable timescales of components in the loop (Novák and Tyson, 2008). Because RA is an essential inducer of spermatogonial differentiation (Morales and Griswold, 1987; van Pelt and de Rooij, 1990), our study reinforces the notion that preleptotenes may supply RA to spermatogonia on a cyclic basis (Raverdeau et al., 2012; Snyder et al., 2011; Sugimoto et al., 2012). The model reproduces *Stra8*-null mouse morphology by altering the differentiation time and lifespan of preleptotenes and leptotenes. Importantly, the model is capable of simulating many other spermatogenic defects and revealing underlying mechanisms. For example, a reduction in the spermatogonial stem cell pool affects all downstream cells including sperm counts.

Additionally, the model was able to interpret the distinctive tubule morphology observed after treatment of mice with WIN 18,446, a promising male contraceptive. The effect of WIN 18,446 can be explained by the arrested differentiation of spermatogonia. The transient increase in progenies following treatment can be explained by the space constraint. When a cell divides, it creates a new cell that will occupy a neighboring micro-compartment. When a cell moves, it will occupy a neighboring micro-compartment. Contact inhibition prevents cell division and movement when all the adjacent sites are filled. On the other hand, empty tubule space following WIN 18,446 treatment can improve the success of division and movement. After the tubule restores its typical number of germ cells, spermatogenesis returns to normal. The model predicts that the long-term outcome after cessation of WIN 18,446 treatment is healthy tubules with normal spermatogenic cycles. We further arrested spermatogonial transition for 16, 24, and 32 days, respectively, to mimic long-term WIN 18,446 treatment, which has not been performed experimentally. Our results indicate that longer treatment leads to more missing cycles. However, the tubule always regains its normal cyclic behavior after treatment cessation.

The current 2D model focuses on the dynamics of meiocytes; stages prior to the onset of meiosis are simplified. For example, only spermatogonial stem cells but not other types of undifferentiated spermatogonia are captured, and A_1_–B spermatogonia are collectively treated as differentiating spermatogonia. Future inclusion of individual agents for all different stages of spermatogonia should allow detailed studies of spermatogonial dynamics. In addition, as new data emerge, the current model can serve as a template on which to add other cells, cytokines, hormones, and molecules to determine how each element augments or abrogates system dynamics. The nuclear morphology of germ cells can be further captured to distinguish different stages. In addition, the model can be extended to 3D to elaborate the process of spermatogenic waves along the testicular tubule (Hogarth and Griswold, 2010). Because testicular tubules consist of a mixture of all 12 stages at one time point, a 3D model can truly relate the testicular morphology to the sperm output. For example, 8-day WIN 18,446 treatment may arrest spermatogonial differentiation for a range of 1–8 days depending on the stage when the treatment starts. A 3D model could also be able to capture all six divisions of differentiating spermatogonia prior to the preleptotene stage. Finally, such a modeling approach may also be applicable to human spermatogenesis, and, hence, may lay the foundation for increasing the effectiveness of male fertility regulation through long-term simulations.

## Supplementary Material

Supplementary Material
