# Supplementary material for: Computer simulations of the mouse spermatogenic cycle
Source: Biol Open. 2014 Dec 12;4(1):1–12. doi: 10.1242/bio.20149068 (PMC4295161; doi:10.1242/bio.20149068)
Supplement: Supplementary Material [file supp_4_1_1__index.html]

Computer simulations of the mouse spermatogenic cycle — Supplementary Material 

# Computer simulations of the mouse spermatogenic cycle

## bio.20149068 Supplementary Material

**Files in this Data Supplement:**

- Supplementary Material - Debjit Ray et al. doi: 10.1242/bio.20149068
- Movie 1 - **Time-lapse simulations of four spermatogenic cycles starting from Stage I in the wildtype mouse.**
- Movie 2 - **Tracing the progenies of one spermatogonial stem cell over four spermatogenic cycles.** A total of 13 elongated spermatids are produced from the stem cell, with 10 being released into the lumen and three undergoing apoptosis.
- Movie 3 - **A time-lapse movie to simulate the progression from normal spermatogenesis to the VAD phenotype.** Four spermatogenic cycles are illustrated starting from Stage I. The preleptotene threshold required for asymmetric division of spermatogonial stem cell is changed from 5 (baseline value) to 50.
- Movie 4 - **A time-lapse movie to simulate four spermatogenic cycles starting from Stage I in the *Stra8*-deficient mice.** Differentiation time of preleptotene is changed from 44 (baseline value) to 93 hours.
- Movie 5 - **A time-lapse movie to simulate WIN 18,446-treated mouse testes.** Six cycles of spermatogenesis are simulated, corresponding to 52 days in real time. Stage I is the initial condition of the simulation. The eight-day treatment is represented in the model as arresting the asymmetric division of spermatogonial stem cells for eight days starting from day five and accelerating the division time of differentiating spermatogonia from 88 (baseline value) to 68 hours.
