# Supplementary material for: Computer simulations of the mouse spermatogenic cycle
Source: Biol Open. 2014 Dec 12;4(1):1–12. doi: 10.1242/bio.20149068 (PMC4295161; doi:10.1242/bio.20149068)
Supplement: Supplementary Material [file supp_bio.20149068_bio.20149068-s1.pdf]

Supplementary Material
Debjit Ray et al. doi: 10.1242/bio.20149068

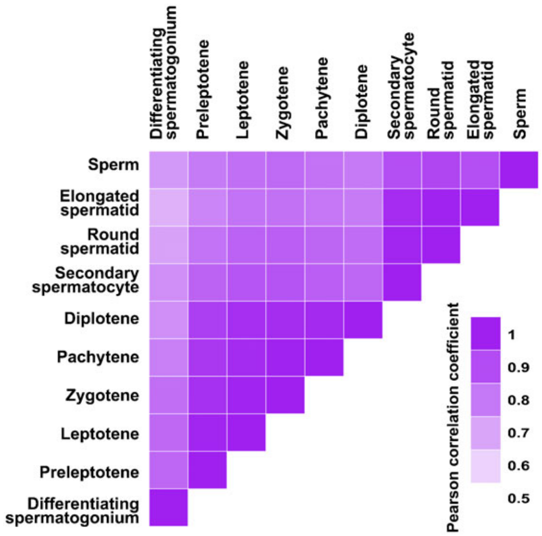

Fig. S1. Pearson correlation coefficients for all pairs of germ cell numbers. For each germ cell type, average cell counts are determined across one spermatogenic cycle from three simulation runs. The Pearson correlation coefficient is calculated for every pair of germ cell numbers over 20 cycles. Cell types originating from the same progenitors are correlated. For example, the numbers of differentiating spermatogonia in cycles 1–20 correlate with the numbers of elongated spermatids in cycles 4–23. As the number of spermatogonial stem cells does not change during simulation, this cell type is not included in the analysis.

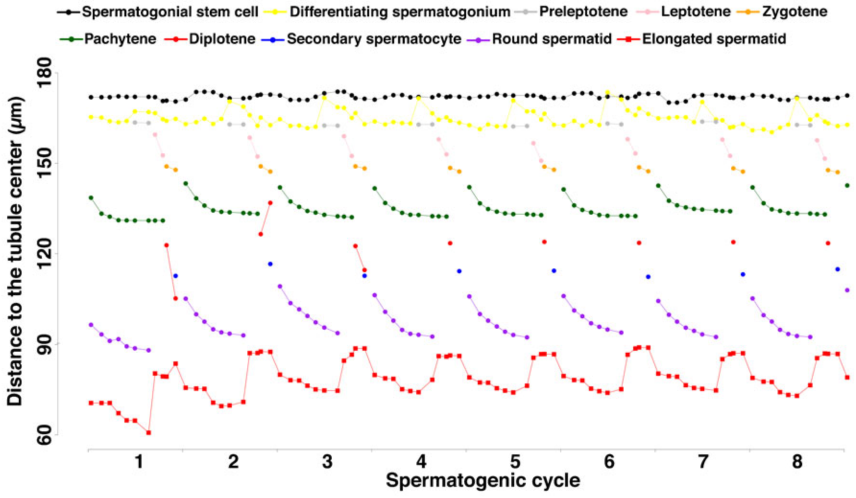

Fig. S2. Positions of germ cells over eight spermatogenic cycles. Stage I is the initial condition of the simulation. The average from three simulation runs is presented.

Biology Open

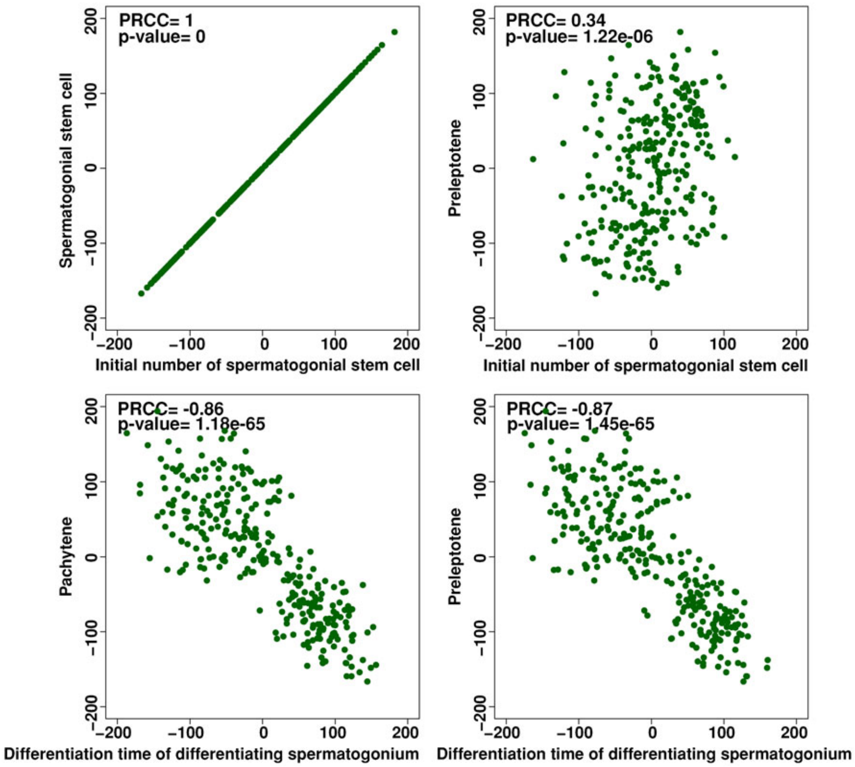

Fig. S3. Examples of PRCC scatter plot. The *x*-axis denotes the residuals of the linear regression between the rank-transformed values of the parameter under investigation versus the rank-transformed values of all the other parameters. The *y*-axis denotes the residuals of the linear regression between the rank-transformed values of the average number of cells across one spermatogenic cycle versus the rank-transformed values of all the other parameters. Parameter sampling is performed 300 times, and PRCC correlates the two residuals shown on the *x*- and *y*-axes.

Table S1. Movement rules for germ cells

		Direction (%)‡							
	Position domain*	F	FL	FR	L	R	B	BL	BR
Spermatogonial stem cell	13–14	50	5	5	0	0	30	5	5
Differentiating spermatogonium	12–14	0	5	5	45	45	0	0	0
Preleptotene	12–14	34	16.5	16.5	16.5	16.5	0	0	0
Leptotene	11–13	34	16.5	16.5	16.5	16.5	0	0	0
Zygotene	9.5–13	34	16.5	16.5	16.5	16.5	0	0	0
Pachytene	9.5–12	60	20	20	0	0	0	0	0
Diplotene	7–12	60	20	20	0	0	0	0	0
Secondary spermatocyte	7–12	60	20	20	0	0	0	0	0
Round spermatid	6–9	34	16.5	16.5	16.5	16.5	0	0	0
Elongated spermatid	4–7	60	20	20	0	0	0	0	0

*The radii of the cross-section and lumen are 14 and 4 micro-compartments, respectively. The 1st layer of micro-compartments is in the center while the 14th layer of micro-compartments is closest to the basement membrane. For example, spermatogonial stem cells are constrained in the 13th–14th layers of micro-compartments.

‡A cell can move into one of the eight adjacent micro-compartments (F: forward; L: left; R: right; B: backward). The probability of moving in eight directions is summed to 1 for each cell type. For spermatogonial stem cells, the forward direction is toward the nearest interstitium. For other cell types, the forward direction is defined as moving toward the lumen.

Biology Open (2015) 000, 1–12 doi:10.1242/bio.20149068

Table S2. Kinetic parameters‡ and initial conditions used in model simulations

Parameter	Value	Reference
Differentiation time of differentiating spermatogonium	*205 hours	(Oakberg, 1956b; Oakberg, 1957)
Differentiation time of preleptotene	*44 hours	(Oakberg, 1956b; Oakberg, 1957)
Differentiation time of leptotene	*24 hours	(Oakberg, 1956b; Oakberg, 1957)
Differentiation time of zygotene	*42 hours	(Oakberg, 1956b; Oakberg, 1957)
Differentiation time of pachytene	*165 hours	(Oakberg, 1956b; Oakberg, 1957)
Differentiation time of round spermatid	*140 hours	(Oakberg, 1956b; Oakberg, 1957)
Division of spermatogonial stem cell	5 preleptotene	(Snyder et al., 2011), estimated
Division time of differentiating spermatogonium	*88 hours	(Nakagawa et al., 2010; Yoshida et al., 2007)
Division time of diplotene	*20 hours	(Oakberg, 1956b; Oakberg, 1957)
Division time of secondary spermatocyte	*22 hours	(Oakberg, 1956b; Oakberg, 1957)
Minimum–maximum lifespan of differentiating spermatogonium	*206–206 hours	(Klein et al., 2010; Nakagawa et al., 2010), estimated
Minimum–maximum lifespan of preleptotene	*43–93 hours	(Bellvé et al., 1977b; Jeyaraj et al., 2003), estimated
Minimum–maximum lifespan of leptotene	*23–73 hours	(Bellvé et al., 1977b; Jeyaraj et al., 2003), estimated
Minimum–maximum lifespan of zygotene	*41–91 hours	(Bellvé et al., 1977b; Jeyaraj et al., 2003), estimated
Minimum–maximum lifespan of pachytene	*164–214 hours	(Bellvé et al., 1977b; Jeyaraj et al., 2003), estimated
Minimum–maximum lifespan of diplotene	*21–23 hours	(Bellvé et al., 1977b; Jeyaraj et al., 2003), estimated
Minimum–maximum lifespan of secondary spermatocyte	*22–25 hours	(Bellvé et al., 1977b; Jeyaraj et al., 2003), estimated
Minimum–maximum lifespan of round spermatid	*139–189 hours	(Bellvé et al., 1977b; Jeyaraj et al., 2003), estimated
Minimum–maximum lifespan of elongated spermatid	*200–230 hours	(Bellvé et al., 1977b; Jeyaraj et al., 2003), estimated
Movement time of spermatogonial stem cell	*60 hours	(Oakberg, 1956b; Oakberg, 1957), estimated
Movement time of differentiating spermatogonium	*10 hours	(Oakberg, 1956b; Oakberg, 1957), estimated
Movement time of preleptotene	*40 hours	(Oakberg, 1956b; Oakberg, 1957), estimated
Movement time of leptotene	*10 hours	(Oakberg, 1956b; Oakberg, 1957), estimated
Movement time of zygotene	*40 hours	(Oakberg, 1956b; Oakberg, 1957), estimated
Movement time of pachytene	*20 hours	(Oakberg, 1956b; Oakberg, 1957), estimated
Movement time of diplotene	*5 hours	(Oakberg, 1956b; Oakberg, 1957), estimated
Movement time of secondary spermatocyte	*5 hours	(Oakberg, 1956b; Oakberg, 1957), estimated
Movement time of round spermatid	*20 hours	(Oakberg, 1956b; Oakberg, 1957), estimated
Movement time of elongated spermatid	*60 hours	(Oakberg, 1956b; Oakberg, 1957), estimated
Initial number of spermatogonial stem cell	*8	(Oakberg, 1956a), newly generated
Initial number of differentiating spermatogonium	12	(Oakberg, 1956a), newly generated
Initial number of pachytene	48	(Oakberg, 1956a), newly generated
Initial number of round spermatid	90	(Oakberg, 1956a), newly generated
Initial number of elongated spermatid	90	(Oakberg, 1956a), newly generated

‡Kinetic parameters represent timers associated with each cell. A timer decreases by one after each hour of simulation. A cellular behavior is triggered once the timer reaches zero.
*Parameters included in the parameter sensitivity analysis.

Table S3. Individual parameter changes leading to the VAD phenotype

Parameter	Change
Initial number of spermatogonial stem cell	↓
Division of spermatogonial stem cell	↑
Differentiation time of differentiating spermatogonium	↑
Division time of differentiating spermatogonium	↑
Lifespan of differentiating spermatogonium	↓
Differentiation time of preleptotene	↓

Table S4. Individual parameter changes leading to the *Stra8*-deficient phenotype

Parameter	Change
Differentiation time of preleptotene	↑
Lifespan of preleptotene	↓
Differentiation time of leptotene	↑
Lifespan of leptotene	↓
Lifespan of zygotene	↓

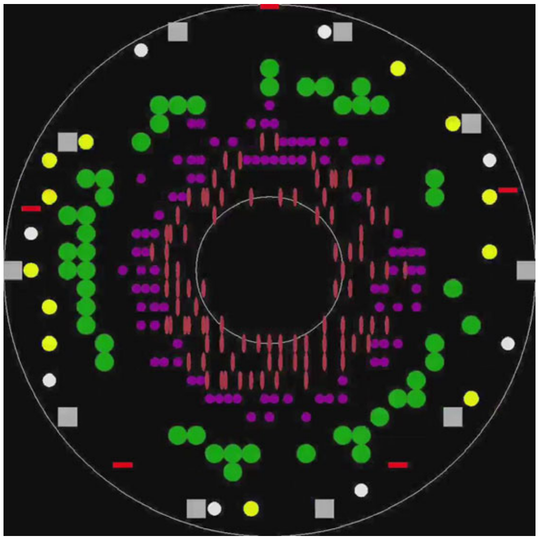

Movie 1. Time-lapse simulations of four spermatogenic cycles starting from Stage I in the wildtype mouse.

Biology Open (2015) 000, 1–12 doi:10.1242/bio.20149068

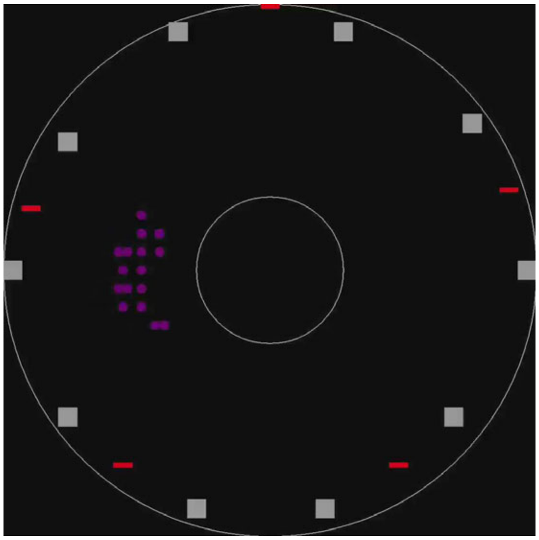

Movie 2. Tracing the progenies of one spermatogonial stem cell over four spermatogenic cycles. A total of 13 elongated spermatids are produced from the stem cell, with 10 being released into the lumen and three undergoing apoptosis.

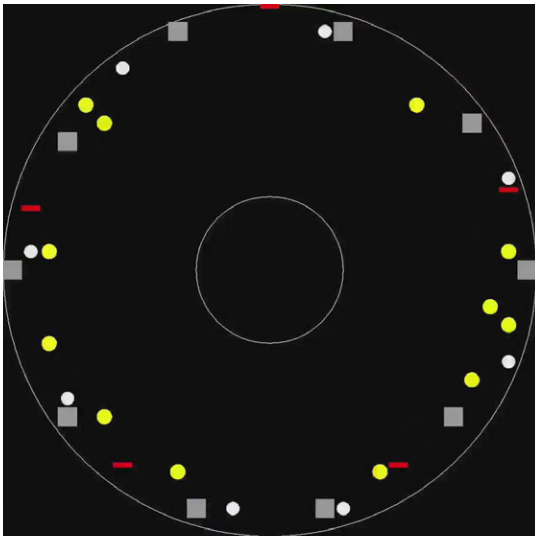

Movie 4. A time-lapse movie to simulate four spermatogenic cycles starting from Stage I in the *Stra8*-deficient mice. Differentiation time of preleptotene is changed from 44 (baseline value) to 93 hours.

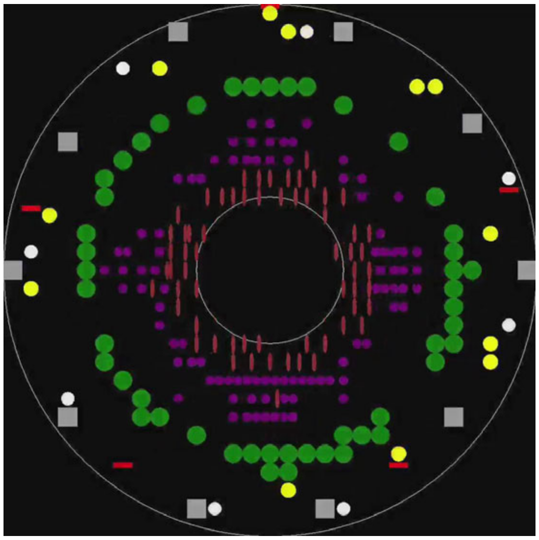

Movie 3. A time-lapse movie to simulate the progression from normal spermatogenesis to the VAD phenotype. Four spermatogenic cycles are illustrated starting from Stage I. The preleptotene threshold required for asymmetric division of spermatogonial stem cell is changed from 5 (baseline value) to 50.

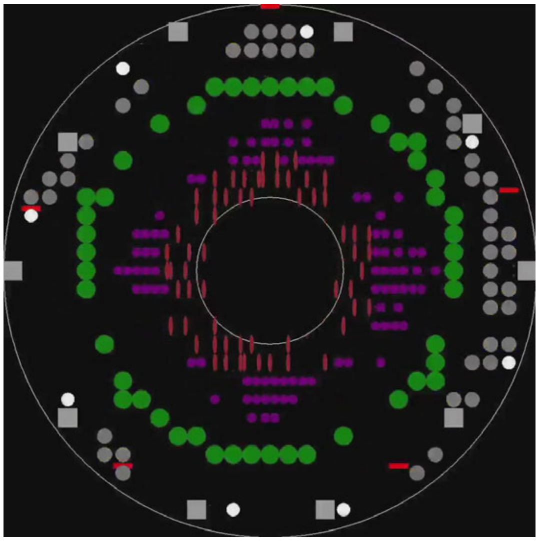

Movie 5. A time-lapse movie to simulate WIN 18,446-treated mouse testes. Six cycles of spermatogenesis are simulated, corresponding to 52 days in real time. Stage I is the initial condition of the simulation. The eight-day treatment is represented in the model as arresting the asymmetric division of spermatogonial stem cells for eight days starting from day five and accelerating the division time of differentiating spermatogonia from 88 (baseline value) to 68 hours.
